# Transient Neonatal Uterine Bleeding Offers a Window to Understanding Uterine Development

**DOI:** 10.3390/biom16101378

**Published:** 2026-09-22

**Authors:** Marwan Habiba, Sun-Wei Guo, Giuseppe Benagiano

**Affiliations:** 1 Department of Health Sciences, University of Leicester, Lancaster Road, Leicester LE1 7RH, UK; 2 University Hospitals of Leicester, Infirmary Square, Leicester LE1 5WW, UK; 3 Department of Biochemistry and Molecular Biology, Research Institute, Shanghai Obstetrics and Gynecology Hospital, Fudan University, Shanghai 200011, China; 4 Faculty of Medicine and Surgery, “Sapienza” University of Rome, 00161 Rome, Italy; 5 Geneva Foundation for Medical Education and Research, 1202 Geneva, Switzerland

**Keywords:** neonatal uterine bleeding, early-onset endometriosis, endometriosis, endometrium, endometrial development

## Abstract

The phenomenon of neonatal uterine bleeding was observed over centuries. It was dismissed as having no clinical relevance. Alternatively, it was hypothesized to be associated with early-onset endometriosis, but its exact clinical significance remains unknown. We conducted a narrative review using an updated literature of Medline database to July 2026. We examined all identified articles on NUB and examined recent controversies. We explored the relevance of the phenomenon to early-onset endometriosis. We highlight NUB as a putative marker for endometrial maturation and development. The incidence of NUB varies with gestational age. The occurrence of bleeding originating in the uterus of some neonates is well documented. It is possible that some instances of bleeding may not be uterine in origin. The fetal and neonatal endometrium differ significantly from that of the adult. Thus, uterine bleeding in the neonate is not analogous to menstruation. While the factors affecting fetal endometrial development are largely unknown, the occurrence of bleeding in some, but not all, neonates call for a better understanding of the uterine development and the underlying mechanisms of bleeding. The observation that some, but not all, neonates exhibit bleeding may have implications on future uterine development and function. The hypothesis that NUB is associated with early-onset endometriosis, while plausible, has no proven evidence but is difficult to falsify. Whether or not an association exists between NUB and early-onset endometriosis, the phenomenon warrants focus on the physiological stages of early uterine development and maturation and how these may be affected by in utero and early neonatal events.

## 1. Introduction

Although the presence of bleeding in female newborns and young babies fascinated writers for over a millennium, it was not till more recently that reports drew a clear distinction between neonatal bleeding, early menstruation due to precocious puberty, and hemorrhage due to disease [1]. Over the centuries, various descriptions were made and a variety of interpretations—with wide contrast between them—were presented. The controversy over its meaning and possible consequences continues even today.

### 1.1. Historical Background

The oldest reference that we were able to identify to vaginal bleeding in a neonate or infant is contained in the Code of Halakha (Jewish religious law) authored by the medieval Sephardic Jewish philosopher Rabbi Moshe ben Maimon (Maimonides) towards the end of the 12th century [2]. In this Code, the Rabbi states that laws of ritual impurity after menstruation apply also to a newborn infant girl. He also mentioned that this requirement is founded on Chapter XII, verses 1–5 of the Bible Book of Leviticus, that—in mentioning ‘Purification After Childbirth’—makes a distinction between a woman who gives birth to a boy and a girl.

The first mention of what we today call Neonatal Uterine Bleeding (NUB) in the Western literature has been attributed to Albertus Magnus in the 13th century. The author listed menstruating since birth as one of three circumstances capable of giving rise to ‘monsters’ [3,4]. At the beginning of the 17th century the French midwife Louise Bourgeois—who published the first obstetrics book to include anatomical data—mentioned the case of ‘a girl who, soon after birth experienced a moderate flux coming from the uterus lasting some ten days’ [5,6].

Other references exist in the classic literature of cases of early bleeding, but there was often confusion between bleeding in the newly born and bleeding due to precocious puberty [1]. There were even those who did not believe that this phenomenon was real. In the 19th century, Nägele was amongst the authors skeptical of the occurrence of such bleeding [7].

The first clear description of NUB was presented by Carus [8] in the early 19th century. He detailed the case of a normal female baby who released mucus from the vagina on day 4 after birth; this was followed by the passage of drops of blood that lasted for a few days. The baby was quite healthy during this process. A few years later, Kahleis [9], described a second case: that of an 8-day old girl with a history of vaginal blood loss. Other references exist in the literature of cases of early bleeding, but confusion continued between bleeding in the newly born and precocious puberty [1]. Early observations commonly featured mucoid secretion followed by bloody discoloration and a small amount of blood at the vulva. The bleeding is initially bright red but becomes dark brown [10]. The description which is often of a healthy neonate helped foster the notion of NUB as a fleeting, medically and scientifically uninteresting feature.

The first detailed description and review of NUB was published in 1876 by Cullingworth [11] who wrote: ‘There occurs now and then in the female infant a discharge from the genital organs which is more or less similar in character to the menstrual fluid, and which makes its appearance within a few days after birth, most commonly, indeed, within the first week. The duration of the flow is generally from two to five days, although occasionally it has been considerably longer, and the quantity, which is sufficient to cause a stain of some size upon the napkin, amounts altogether to what would be considered an ordinary menstrual flow proportioned to the child’s tender age and diminutive size.”

This was followed by a number of case descriptions and interpretations of NUB and its meaning, but during the 20th century there was a progressive disinterest in NUB, since it was considered of no clinical relevance. The situation changed when Brosens and Benagiano [12] first proposed that NUB might be causally involved in early-onset endometriosis (EOE).

### 1.2. More Recent Developments

Perhaps stimulated by the possibility of an association between NUB and endometriosis, work followed and the hypothesis was tested and challenged. In a retrospective case-controlled web-based questionnaire study, Ogawa et al. [13] recruited 1093 women born between 1996 and 2000 and 807 born between 2013 and 2017 who had symptoms suggestive of endometriosis. The incidence of NUB was 9.6% (n = 105) in the first and 3.1% (n = 25) in the second cohort. Multiple regression analysis identified younger maternal age and gestation of >39 weeks to be significantly associated with NUB. Although there is a possibility of recall bias, newborn females who had a history of NUB had a higher risk of complaining of endometriosis-related symptoms in adult life [13].

In another clinico-pathological study, Ogawa et al. [14] retrospectively collected 15 postmortem neonatal endometria and prospectively collected blood from nappies of 18 female babies. They analyzed a range of biological markers. They concluded that they did not find any supporting evidence for the role of NUB in EOE and that future studies with large sample size and application of modified technological tools are warranted to test the NUB hypothesis and to clarify its biological or clinical significance. In view of these findings, Habiba et al. [15] carried out a full review of the potential role of NUB in EOE and discussed factors that argue against the existence of a link between EOE and NUB. Habiba et al. [15] concluded that a link is plausible but that finding supporting evidence will be very challenging because there is, as yet, no proof of: (a) the existence of viable endometrial cells or endometrial mesenchymal stem cells in NUB; (b) the passage of these cells into the pelvic cavity; (c) their possible response to steroids; (d) whether they can reside within the pelvic cavity and remain dormant till menarche without being removed by immune cells (Figure 1).

Leaving aside this hypothesis and the ensuing debate, a further evaluation of the phenomenon remains important, since it offers the opportunity to further our understanding of the status of the uterine mucosa around birth. For this reason, we carried out a new search of existing data about NUB and assessed the literature through a parallel lens of endometrial development.

## 2. Material and Methods

As already published, in December 2023 [15] we undertook a Medline search using the terms “premenarcheal endometriosis”, or “early-onset endometriosis” (n = 12), “neonatal uterine bleeding”, or “neonatal menstruation” (n = 18), and neonate or newborn plus uterine bleeding or hemorrhage plus endometriosis (n = 23). Identified articles were combined (n = 37) and searched manually to select all articles that addressed the relationship between NUB and endometriosis (n = 26). The latter were reviewed in full text to extract relevant information. We also undertook a search using the terms “adolescent” or “pre-menarche” or “menarche” (n = 2,279,914) and “endometriosis” (n = 34,073). These were combined yielding 2264 articles, which in turn were combined with the search terms “pathophysiology” and “etiology or cause”, yielding 196 articles that were searched based on title and abstract, and 21 were selected for full-text review. On the 1 July 2026, we undertook an updated search covering the period since December 2023. The updated search identified 3 additional articles. Our historical assessment comprised a synopsis from our previous review of the history of NUB [1].

## 3. Results

We here address pathophysiology and frequency of occurrence in so far as it is relevant to understanding the phenomena and examine its possible clinical relevance including the hypothesis of a link to EOE.

### 3.1. Pathogenesis

As mentioned, the first ‘scientific’ approach to the study of NUB was undertaken towards the end of the 19th century by Cullingworth [11] who collated cases that were available to him that were published up to that date. He concluded that its occurrence was not the consequence of a pathological event. He wrote: “No case of hemorrhage from the female genital organs in the newly born has hitherto been recorded in which the general health had been seriously impaired thereby, and still less which has been attended with a fatal result.” Subsequent early observations agreed on the fact that bleeding is often preceded by mucoid secretion and bloody discoloration. The amount of blood that appeared afterwards was small. The bleeding, which is initially bright red, becomes dark brown or chocolate colored. Schukowsky [10], who was the first to observe the blood under the microscope, concluded that it showed no abnormality and Kouindjy [19] reported two cases of NUB in consecutive pregnancies of the same woman.

It is notable that Cullingworth [11] was aware that not all his contemporaries were convinced that NUB is unique to females and that he discussed the view expressed by Dr Camerer that the phenomenon results from tying the umbilical cord. Camerer’s view, as considered by Cullingworth, was that the corresponding bleeding in the male infant is gastrointestinal melena.

Worthy of mention is that as late as 1901, Williams [20] wrote that in a few instances, bleeding “merge into regular menstruation, generally with the appearance of other manifestations of precocious sexual development”. This means that even at the beginning of the 20th century confusion persisted between NUB and precocious puberty and that no attempt was made at a pathophysiological evaluation of the phenomenon.

There was, however, one major exception: In 1904, the Austrian obstetrician and gynecologist Josef von Halban examined neonatal uteri collected at autopsy and identified instances of endometrial breakdown and microscopic blood extravasation. In some cases, there were histological features of stromal vascular congestion and blood extravasation. Halban was the first to present evidence that around month 8 of gestation a series of phenomena occur in the fetal uterine mucosa leading to significant hyperemia and changes which he described as analogous to those that occur during the menstrual stage in adults. He stressed that these modifications are not uniform and can occur to varying degrees and encompass “all the stages of pre-menstrual hyperemia, up to sub-epithelial hemorrhages and free blood leakage into the uterine cavity.” Importantly, he observed that “almost always blood can be detected only with the microscope” [16,17]. It must be noted that Halban believed that even the full reaction causing uterine bleeding represented a physiological, not a pathological, process.

Halban provided another important insight that must have been considered revolutionary at the time when he wrote: “these changes must be interpreted as a reaction to active substances present during pregnancy. While the maternal uterus reacts with decidua formation, the weaker fetal uterus reaction leads only to menstrual-like changes.” Halban went on to state that these active placental substances are “some kind of internal secretion from this organ” and that they are then “secreted in the same way to both mother and fetus” [17]. In summary, Halban believed that the placenta produced substances able to influence the fetus and specifically the endometrium with different degrees of intensity. The observation led him to conclude that there were various stages of endometrial maturation around birth. This early observation may explain why NUB is observed in only a small percentage of newborns.

It took 50 years for other researchers to “rediscover” that it is only during the last month of pregnancy that the fetal endometrium features evidence of reacting to steroid action [21] and that in the majority of near-term fetuses the endometrium does not respond to the very high levels of circulating progesterone [22].

As mentioned above, although the phenomenon of NUB has been observed over the centuries, little curiosity was raised about its nature and genesis. Philipp [23] referred to it in terms of ‘die bekannten Vaginalblutungen der Neugeborenen’ (the well-known vaginal bleeding of newborns), which indicates that the observation was seen as normal. Kaiser [24], followed from the observations made sixty years earlier by Halban [16] and demonstrated hormonal changes in the fetal endometrium in the second and third trimesters of pregnancy. Kaiser [24] documented that the endometrium in the 8th month appears tall and cylindrical, with multi-layered nuclei and distinct basal vacuoles. His view was that the uterine lining undergoes involution and that it regresses to short invaginations about day 14 after birth. Kaiser argued that the occasional bleeding is the result of diapedesis, not desquamation [24].

### 3.2. Is NUB Associated with Early-Onset Endometriosis?

While retrograde menstruation remains the most widely recognized theory for endometriosis, rare cases have been reported before menarche which poses a question about the source of the endometrial deposits. NUB, both visible and occult, are true observed phenomena, and the neonatal uterus features a long-closed cervix which can increase the likelihood of retrograde flow. These observations raised the question whether NUB can be the source of stem cells that can remain dormant till pre-menarche. The observations formed the basis for the hypothesis that NUB may be linked to early-onset endometriosis [12]. It is important to reiterate that the hypothesis, which seeks to explain early-onset endometriosis with reference to Sampson’s theory of retrograde menstruation, remains speculative with no direct proof. There is currently no direct evidence demonstrating any aspect of the sequence from neonatal bleeding to viable cell reflux, pelvic implantation, prolonged dormancy, or subsequent development of endometriosis.

An important contribution to this field was provided by the study by Ogawa et al. [14]. Their study included 15 postmortem neonatal endometrial biopsies and blood samples collected from diapers from 18 cases of NUB. The samples were analyzed for ovarian steroid receptors [estrogen receptor (ER), progesterone receptor (PGR)], and markers of: (a) decidualization: prolactin (PRL), and insulin growth factor binding protein 1 (IGFBP1)]; (b) pre-decidualization: Glycodelin A (Gd-A), α-smooth muscle actin (a-SMA); (c) proliferation: Ki-67 index; (d) cluster of differentiation (CD): CD31+ cells, immunocompetent CD68+, CD45+, CD56+ cell; (e) some putative markers of endometrial mesenchymal stem cells (eMSCs). The autopsies ranged from gestational week 27 to 40 and included one stillbirth at 34 weeks. Neonatal deaths occurred from age 0 to 32 days after birth. Histological examination revealed secretory changes in all but two cases which featured proliferative endometrium. The two instances of proliferative endometrium were in babies delivered at 37 and 38 weeks. There was variable expression of some phenotypes of eMSCs (CD90/CD105) in neonatal endometrial, but no eMSCs or endometrial cells were identified in the samples of NUB that were collected. Whilst this casts doubt on the role of NUB as a mechanism of early-onset endometriosis, it demonstrates that the endometrium, including in cases delivered at 27 weeks, featured ‘secretory’ response. It is notable that secretory changes were still observed in endometria at 10, 12, 17, 18, and 32 days postnatally. It is also interesting that a few red blood cells were detected in the uterine stroma in three cases (20%) and hemorrhage in the endometrium was found in two cases (13.4%) one of which featured proliferative endometrium (died day 8) and the other showed secretory changes (died day 1). This can be taken to indicate that bleeding may occur in the absence of secretory changes [14].

The long-recognized discoloration seen on nappies of newborns (e.g., [25,26]) came into renewed focus with the publication by Fornelli et al. [18]. They conducted a cross-sectional prospective study aimed to address the nature and correlates of NUB. This included laboratory analysis that compared extracts from diapers from female and a control group of male neonates. Non-dried material was obtained from the diapers using a spatula and diluted in 50 mL TRIS buffer and then examined microscopically. The study also used the Seratec PMB test to distinguish menstrual from peripheral blood. Reddish staining was reported in 22% of female and 13% of male neonates. Since only 2 (2.5%) females were positive for menstrual blood, Fornelli et al. attributed the discoloration to the presence of urate crystals. Peripheral blood was detected in 8/78 (10.3%) of collected female diapers and menstrual blood in 2/78 (2.5%). Peripheral blood was also identified in 7/68 (10.3%) of male diapers. The authors concluded that the finding of reddish discoloration on diapers is related to neonatal urate [18]. The study raises a number of interesting points. Positivity for the test for adult menstrual blood was found in 2.5% of female neonates which is a small percentage but, given the well-recognized lack of maturation of the neonatal endometrium, is important. The observation raises a relevant question about the mechanism of such bleeding. It is notable that Kaiser [24] was unable to identify shed endometrium in the cases he examined, yet he remained convinced that the bleeding was uterine. This led him to conclude that the occasional bleeding in female neonates is the result of diapedesis not desquamation. Kaiser’s observation that the neonatal endometrium does not have a mature functionalis may also be relevant to the negative Seratec PMB in the study by Fornelli et al. [24] because it shows that bleeding in the neonate is not analogous to adult menstruation. On the other hand, Seratec PMB test, which relies on the detection of hemoglobin and D-Dimers, is not uniquely specific to menstruation [27]. D-Dimers are 2000 times higher in adult menstrual blood compared to peripheral blood and it is unknown if the same applies to the neonate. This brings into question the applicability of the test to the situation in the neonate. The observation of blood in male infants was commented on in early writings [11]. However, the source of such bleeding in the male remains to be explained.

Fornelli et al. [26] concluded that their finding that reddish discoloration is predominantly caused by urate crystals provides a substantial paradigm shift that redefines the clinical significance of NUB. However, the significance of NUB is not only derived from whether it explains all cases of discoloration of nappies but is also related to the observation that it occurs in a minority rather than in all cases. If the findings of blood with as well as blood without menstrual characteristics are confirmed, this will raise important questions about the mechanisms of bleeding and the different underlying stages of endometrial maturation.

In contrast to the study by Fornelli et al. [26], Ogawa et al. [19] were able to identify erythrocytes, scattered leukocytes and squamous cells in all of the 18 cases they examined. As mentioned above, squamous cells in NUB showed strong positive cytoplasmic reaction to PGR, Gd-A, and Sushi domain-containing protein 2 (SUSD2), but weak or no staining for other markers: epithelial cell adhesion molecule, CD10, ER, PRL, IGFBP1, α-SMA, and/or other eMSCs markers: platelet-derived growth factor receptor beta, CD90, CD105. There were no epithelial/stromal cells and/or eMSCs in NUB. Cases positive for PGR- and PRL-stained squamous cells in NUB also exhibited immunostaining of PGR and PRL in endometria and neonatal vaginal epithelia. CD31-stained micro-vessels in superficial and deeper parts of the neonatal vaginal wall raised the possibility that part of the NUB may originate from the neonatal vaginal wall. On the other hand, Ogawa et al. [19] were able to identify erythrocyte in the samples obtained from NUB and despite microscopic analysis, they did not comment on identifying urate crystals (Figure 2).

### 3.3. The Fetal Endometrium

As mentioned, Halban was the first to report that a series of changes occur in the fetal uterine mucosa that mimic those that occur during the menstrual stage in adult women. Halban also used the expression “neonatal menstruation” to describe NUB and referenced that this expression indicates the presence of an endometrial response in the newborn that is similar to adult menstruation and added that this included features of decidualization [21,22].

Further information became available with the publication by Ober and Bernstein [24]. In this study, the authors evaluated 169 endometria around term. The majority 68.6% (n = 116) were classed as proliferative, and 35.5% (n = 60) were classed as being in the resting phase. The glands were noted to be simple and non-tortuous. Only 10% (n = 17) of the cases featured some degree of gland tortuosity and there was only occasional pseudo-stratified glandular epithelium. Five cases were classed as menstrual endometrium, and these had some secretory feature. There was no correlation between features linked to steroid effect on the ovary and on the endometrium. This clearly shows that the fetal endometrium differs significantly from that of the adult. In the fetus, proliferative endometrium features cuboidal epithelium and simple glands. Secretory features are limited to the appearance of subnuclear and supranuclear vacuoles in glandular epithelium and increased stromal cytoplasmic substance and mosaic architecture is interpreted as decidual changes [24]. The features do not show mature adult type endometrium, stroma or vasculature.

Little is known about the preterm fetal endometrium which is necessarily less developed than that of term neonates. The endometrium could also be affected by associated stressors such as fetal growth restriction, pre-eclampsia and post-maturity. The uterus of very premature neonate has been shown to increase in size in response to exogenous steroids administered after birth. Subsequent steroid withdrawal results in reduction in size [28]. The relevance of in utero growth until term delivery on future uterine function is unknown, but the observation that exogenous steroids have a measurable effect on the postnatal uterus of the preterm neonate provides an opportunity to study the implications on the adult uterus [28]. Fetal growth restriction and post-maturity have both been associated with endometriosis risk as well as a higher incidence of NUB [29,30] (Figure 3).

The effect of maternally administered steroids in the peripartum period is unknown. Preterm delivery entails a period of endometrial development in the absence of the natural maternally derived steroids. How that may affect the endometrium of the adult is unknown, but the uterus continues to develop in preterm infants receiving estrogen and progestogen replacement [31]. In utero steroid exposure has been linked to increased risk of endometriosis and to increased uterine bleeding in the neonate [32].

Ogawa et al. [14] reported that neonatal endometria showed variable expression of ovarian steroid receptors, decidualization, and a substantial amount of proliferative and angiogenic activity. There was also significantly lower tissue accumulation of immunocompetent cells in neonatal endometria. They proposed that this could be a factor that explains the survival of ER+ and PGR+ cells should they make entry into the pelvis. Survival of these cells can, in turn, lead to the development of early endometriosis with the onset of ovarian function [14].

The lack of a developed secretory or decidual endometrium suggests that other mechanisms are responsible for regression of the effects of maternal steroids and the occurrence of bleeding.

The concepts of “ontogenetic progesterone resistance” [31] and “menstrual pre-conditioning” [32] may be relevant to discussions on NUB. These imply that the human uterus starts as a relatively immature organ that acquires the competence of deep placentation in response to dynamic remodeling events triggered by menstruation, miscarriage or parturition.

### 3.4. Frequency

The phenomenon of NUB has attracted much attention and skepticism. In his extensive review of the early literature, Cullingworth [11] mentioned that “any old, experienced nurse will be able to tell that the colored discharge from young girls is an almost daily occurrence.”

In any case, the incidence reported in early publications varied widely. As early as 1835, Billard [33] observed coagulated blood in the uterus of 2 out of 700 autopsies of newborn infants. Then, in 1902, Schukowsky [10] reported the presence of NUB in 0.18% of cases in what probably remains the largest series of observation in neonates (n = 20,000). In his thesis of 1905, Renouf [34] found visible NUB in 2.6% (n = 721) of cases. Zacharias [35] found NUB in 2.5% out of 400 cases. Lisi [36] mentioned that pediatricians and nurses estimated that around 2–3% of newborns experience blood loss with an average duration of 24–48 h.

More recently, Levy et al., reported NUB in 4.5% of 1057 cases [29]. Zubovich reported macroscopic NUB in 7.8% and microscopic NUB in 66% (n = 541) [37]. Kaiser et al. reported visible NUB in 5.3% and when using hemoglobin detection in 61.3% (n = 153) [38]. Huber found visible NUB in 3.3% and occult NUB in 25.4% (n = 350) [39]. The study by Fornelli et al. [18], referred to above, found menstrual blood in 2.5% of their cases.

Of particular interest is the study by Berić et al., who examined 2477 neonates for visible NUB, and analyzed this in relation to gestational age. Bleeding was less common in preterm (0.8%, n = 128) than in term babies (3.8% n = 2241), and was highest in post-term (9.1%, n = 110) babies [40].

### 3.5. Clinical Relevance

Whilst the exact incidence is debatable, there is sufficient evidence that at least some of the red discoloration seen on diapers is indeed a blood stain. There is also evidence, that requires confirmation, that a proportion of this, perhaps small, has similar characteristics to menstrual blood. Thus, the existence of NUB is well-established, and this raises important questions about its mechanism, why it occurs in some but not all neonates, and what significance it might have as an index of uterine maturation.

Various theories have been proposed. Philipp [23] argued that NUB originates from shedding of the entire hypertrophied cervical glandular tissue that occurs just a few days after birth. He also described considerable reduction of vaginal epithelium from 80 to 90 layers to only 5–6 cell layers after birth and argued that this change contrasts with the absent response in the corpus mucosa. Some 20 years later, Kaiser [24] produced evidence that contradicted Philipp as he demonstrated that the endometrium can exhibit secretory features from the 8th month (32nd week) of pregnancy. It is important to reiterate that the term secretory endometrium should not be taken to mean fully developed adult type endometrium. The finding of vaginal epithelial cells was also highlighted in the study by Ogawa et al., who also suggested that some NUB may originate from the vaginal epithelium [14].

It has long been recognized that the fetal endometrium can respond to maternally derived hormones [41], but there remain many points of departure from the features in the adult. On the one hand, the physiological withdrawal of maternally derived steroids that happens after birth provides a plausible reason to expect shedding of the neonatal endometrium. This theory derives from extrapolation from the case of the adult and suggests a type of neonatal menstruation (the term used by Halban). However, it is well recognized that the neonatal endometrium does not exhibit the same mature features in the glands, stroma or blood vessels as the adult. The fetal endometrium does not show the distinction of basalis and functionalis. This raises an important question about the appropriateness of applying tests designed for mature menstruation to the case of NUB. Fetal ‘secretory’ changes have also been shown to persist in some neonates beyond the first few days of life. These are best described as immature secretory features. In addition, some endometria at birth feature ‘proliferative’ changes, which again raises questions about uterine and more specifically endometrial response to steroids and their withdrawal. This also raises questions about the relevance of extrapolating from the adult mechanism of menstruation onto NUB [41]. As already mentioned, Kaiser was unable to identify shed endometrium in cases of NUB and proposed the bleeding occurs through diapedesis [25]. It is indeed possible that mechanisms other than endometrial shedding are responsible for NUB including cases that are negative to Seratec PMB test.

As already mentioned, it has been proposed that NUB might be related to early-onset endometriosis [12,13]. In favor of this hypothesis is the anatomically long cervix, which can represent an obstacle to vaginal flow and can favor retrograde bleeding. On the other hand, the short period of NUB, when contrasted to the much longer period of bleeding during women’s reproductive lifespan, may greatly diminish the chance of seeding of endometrial or stem cells [15]. That this intriguing hypothesis is difficult to falsify is well recognized, but alternative theories exist, including that neonatal bleeding originates in the vagina or cervix and indeed the theory that NUB has no clinical significance.

Ogawa et al. reported the finding of eMSCs with CD90+ and CD105+ phenotypes in neonatal endometrium. In addition, fetal endometrium featured higher expression of prolactin but lower expression of IGFBP1 [14]. Kämmerer et al. [42] demonstrated lower density of endometrial leukocytes (CD45+ and CD68+ cells) in the developing fetal uterus compared to the case in neonates and children. CD56+ and HLA-DR+ cells were absent in the fetus [42]. Thus, the uterus and the endometrium at birth differs from that of the adult and undergoes progressive development postnatally. Extensive animal experiments have shown that the endometrium is readily affected by exogenous steroids [43,44] and that the developing neonatal uterus is sensitive to endocrine disruption.

It is well recognized that individuals born preterm have an increased risk of infertility, miscarriage and pre-eclampsia all of which may be mediated through mechanisms related to the endometrium and, in turn, may be linked to the early stages of development. While much of this awaits proof, exploring the reason(s) why NUB occurs in some, but not all, female neonates may enable better understanding of the stages of endometrial and uterine development. Long-term follow-up studies are needed to assess the clinical significance of NUB. These studies can shed light on the pathogenesis of endometriosis, adenomyosis and the potential impact on future reproductive performance. Ideally, such studies should be prospective and include standardization of diaper collection and sensitive biochemical testing for hemoglobin, blood and urates in the first week of neonatal life and be stratified for gestational age, fetal growth and complications of pregnancy such as diabetes and pre-eclampsia. Efforts should be made to assess any effect of cord clamping and to exclude any other sources of bleeding or contamination and to determine the anatomical origin, the cellular and molecular characterization and the correlation of NUB with gestational age and perinatal exposures. Long-term follow-up will be necessary to address the question of the relation to endometriosis or future menstrual and reproductive function. Studies are also needed that examine fetal and neonatal uteri to further our understanding of the stages of uterine development including the effects of gestational age and of exogenous steroids.

## 4. Discussion

The occurrence of NUB in some but not all neonates, the presence of menstrual type endometrium in a small proportion of neonates, together with the known differences in endometrial features at birth, suggests that this is an important focus for future research. Whilst some findings remain controversial and debate continues about its incidence, the fact that NUB occurs in a small proportion of neonates invites questions about its pathogenesis and clinical significance.

In theory, NUB could result in retrograde flow of endometrial tissue into the peritoneal cavity. This is particularly the case given the relatively long fetal cervix that can offer resistance to antegrade flow. If such bleeding contains stem cells, these may remain dormant till activated with the onset of puberty leading to endometriosis. Since its conception, the authors warned of the difficulty in testing the hypothesis that NUB is linked to EOE [12,13]. However, irrespective of whether NUB is associated with endometriosis, the occurrence of such bleeding in some, but not all, neonates provides, by and in itself, an important invitation to study the phenomenon because of its possible implications beyond endometriosis. This emphasizes the significance of the previously reported differences in histological features at birth and opens an opportunity to assess longer term implications for endometrial function and dysfunction. This includes how the endometrium of term and preterm neonates develops after birth in steroid-free environments and how this might be affected by the administration of potent steroids such as those used to enhance fetal lung maturity. Whilst caution is required from extrapolation from animal studies, it is now well recognized that steroids and their selective modulators do affect mice uterine development leading to adenomyosis. The question must thus be raised about whether factors in fetal or early neonatal life can affect human uterine development and influence future function including the development of adenomyosis and, consequently, whether more attention is needed to optimize the conditions for the developing uterus.

Fornelli et al. [18] argued that their findings challenge the paradigm that NUB represents the first menstrual episode. Indeed, historical data clearly indicate that NUB is not a menstrual episode. Their very data, however, is consistent with the view that some bleeding in the neonate originates in the uterus. The observation that some, but not all, cases with identified blood showed a positive reaction to the Seratec PMB test adds an interesting dimension and invites further research to explain the occurrence.

We argue that the phenomenon of NUB is important, in as far as it is an indicator of the underlying endometrial change. Understanding the occurrence of NUB can further our knowledge of early uterine development, including the effect of prematurity and post-date delivery. Our understanding of early uterine development is in its infancy but the recent finding that first trimester administration of paracetamol, long regarded as safe to use in pregnancy, can affect uterine size in the neonate should act as a further stimulate for research in this area [45].

We must recognize that the current review is limited by the size of the available literature and by the lack of recent large-scale studies that can help address the outstanding questions. This has forced reliance on historical reports, many of which are in the form of bedside observations and have a limited scope beyond the observation of the absence of systematic follow-up. Much of historical research was based on limited case reports and observations that did not employ biochemical tests. In addition, the observation that NUB was not associated with ill-health meant that little attention was paid to the phenomenon. The article is also limited because of the paucity of research on neonatal endometrium, a limitation which we hope that this review would be a stimulus towards remedy.

## 5. Conclusions

The phenomenon of NUB has been subject to controversy since it was first recorded. While the exact incidence and whether it is related to EOE are subject to debate, the occurrence of NUB should invite scientific study of the factors that affect its occurrence and of NUB as an index of endometrial development at term or preterm infants. Advancing our understanding of this neglected field can have an implication on conditions that affect reproductive-age women.

## Figures and Tables

**Figure 1:**
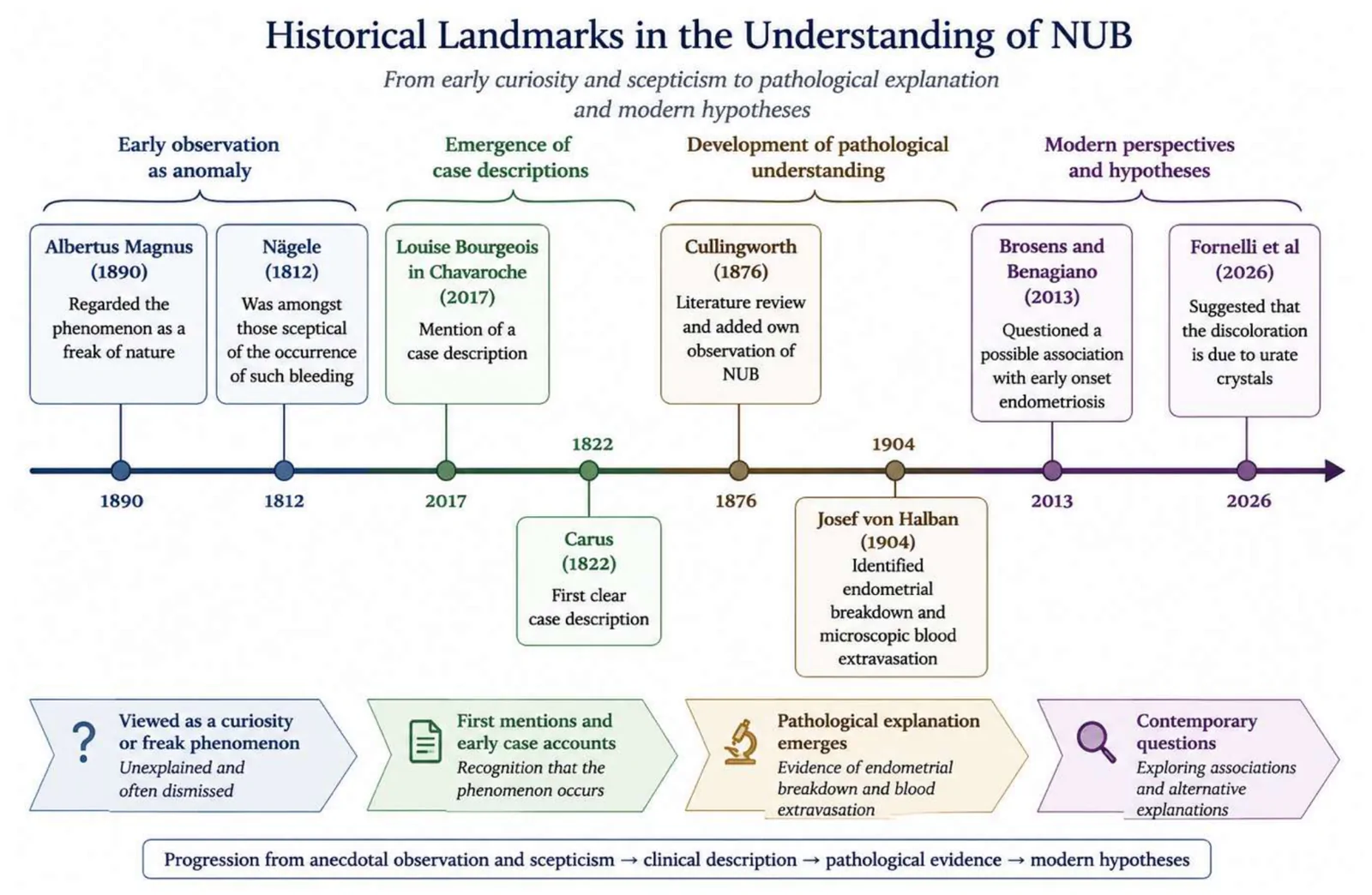
Landmarks in progressive understanding of neonatal uterine bleeding [3,5,7,8,11,12,16,17,18].

**Figure 2:**
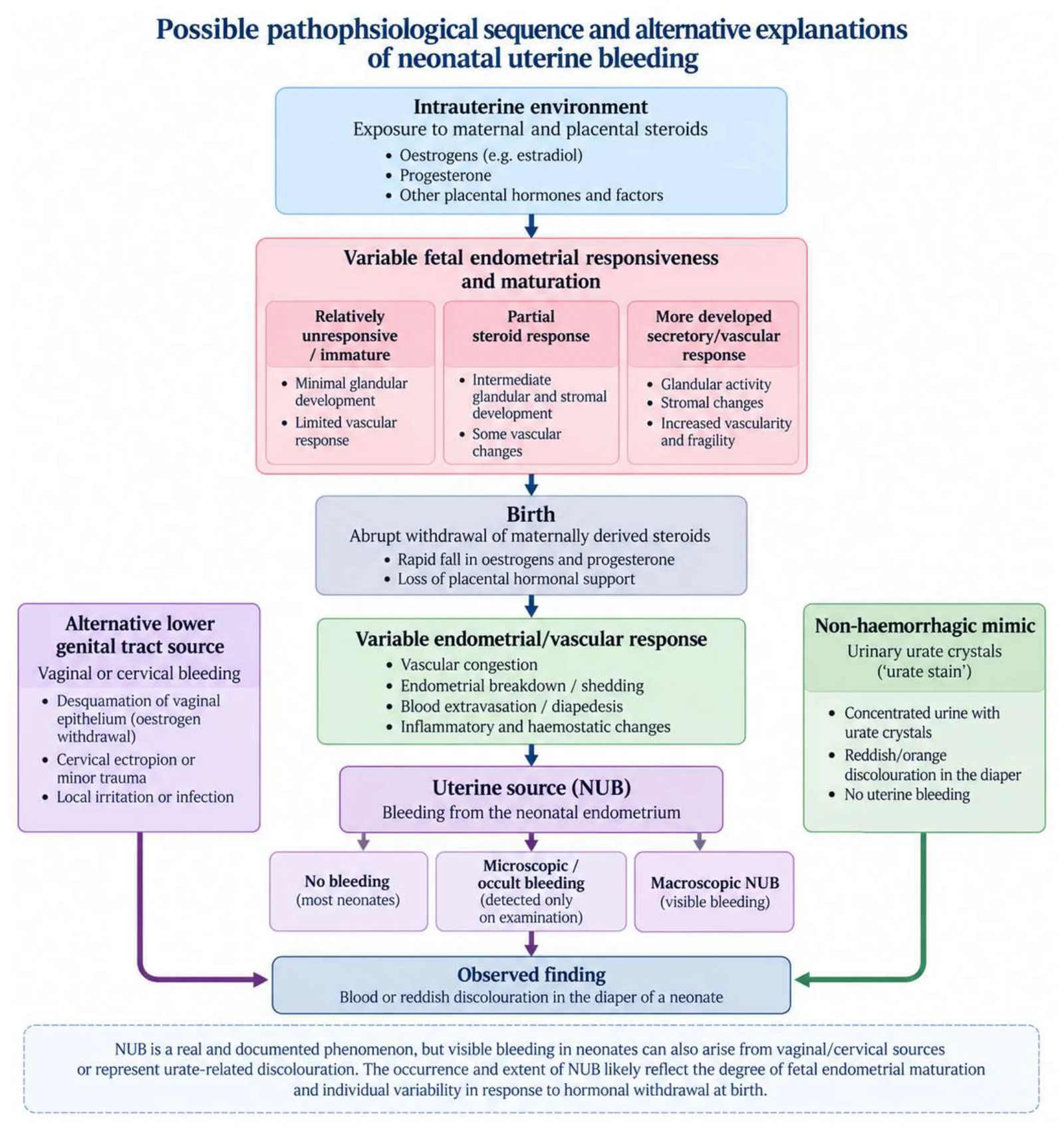
Possible mechanisms and alternative explanations of neonatal uterine bleeding.

**Figure 3:**
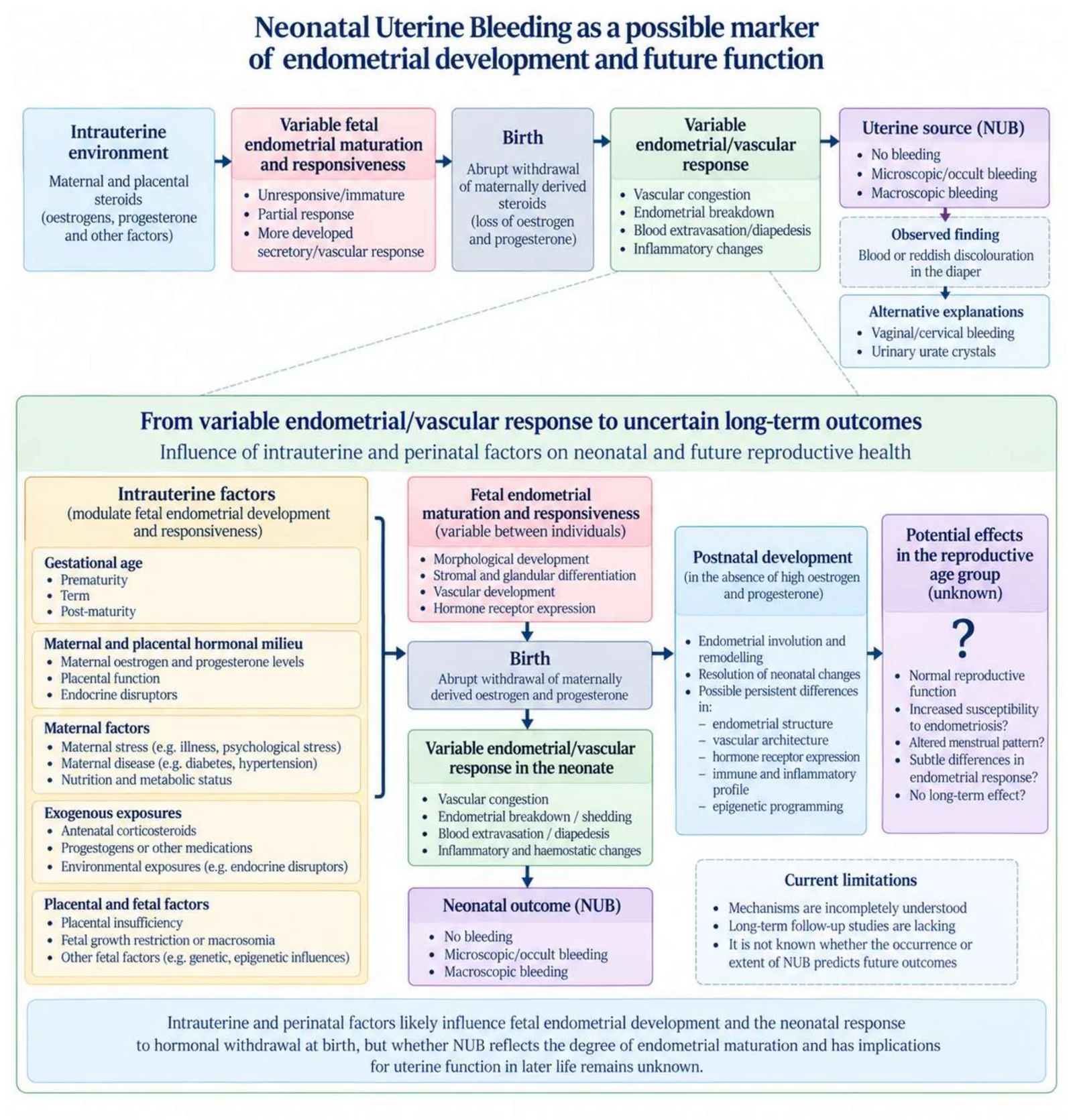
Neonatal uterine bleeding as a possible indicator of current and future uterine development and function.

## Data Availability

No new data were created or analyzed in this study.
